# Thermoplasmatales and Methanogens: Potential Association with the Crenarchaeol Production in Chinese Soils

**DOI:** 10.3389/fmicb.2017.01200

**Published:** 2017-06-30

**Authors:** Fuyan Li, Fengfeng Zheng, Yongli Wang, Weiguo Liu, Chuanlun L. Zhang

**Affiliations:** ^1^Department of Ocean Science and Engineering, Southern University of Science and TechnologyShenzhen, China; ^2^College of Life Sciences, Wuhan UniversityWuhan, China; ^3^Division of Geological and Planetary Sciences, California Institute of Technology, PasadenaCA, United States; ^4^State Key Laboratory of Marine Geology, Tongji UniversityShanghai, China; ^5^Key Laboratory of Petroleum Resources Research, Institute of Geology and Geophysics, Chinese Academy of SciencesLanzhou, China; ^6^State Key Laboratory of Loess and Quaternary Geology, Institute of Earth Environment, Chinese Academy of SciencesXi’an, China; ^7^School of Human Settlement and Civil Engineering, Xi’an Jiaotong UniversityXi’an, China

**Keywords:** methanogens, Thermoplasmatales, Thaumarchaeota, crenarchaeol, Chinese soils

## Abstract

Crenarchaeol is a unique isoprenoid glycerol dibiphytanyl glycerol tetraether (iGDGT) lipid, which is only identified in cultures of ammonia-oxidizing Thaumarchaeota. However, the taxonomic origins of crenarchaeol have been debated recently. The archaeal populations, other than Thaumarchaeota, may have associations with the production of crenarchaeol in ecosystems characterized by non-thaumarchaeotal microorganisms. To this end, we investigated 47 surface soils from upland and wetland soils and rice fields and another three surface sediments from river banks. The goal was to examine the archaeal community compositions in comparison with patterns of iGDGTs in four fractional forms (intact polar-, core-, monoglycosidic- and diglycosidic-lipid fractions) along gradients of environments. The DistLM analysis identified that Group I.1b Thaumarchaeota were mainly responsible for changes in crenarchaeol in the overall soil samples; however, Thermoplasmatales may also contribute to it. This is further supported by the comparison of crenarchaeol between samples characterized by methanogens, Thermoplasmatales or Group I.1b Thaumarchaeota, which suggests that the former two may contribute to the crenarchaeol pool. Last, when samples containing enhanced abundance of Thermoplasmatales and methanogens were considered, crenarchaeol was observed to correlate positively with Thermoplasmatales and archaeol, respectively. Collectively, our data suggest that the crenarchaeol production is mainly derived from Thaumarchaeota and partly associated with uncultured representatives of Thermoplasmatales and archaeol-producing methanogens in soil environments that may be in favor of their growth. Our finding supports the notion that Thaumarchaeota may not be the sole source of crenarchaeol in the natural environment, which may have implication for the evolution of lipid synthesis among different types of archaea.

## Introduction

Since the domain Archaea was proposed in 1977 (Woese and Fox, 1977), their biology, diversity and ecology have been widely studied. Euryarchaeota and Thaumarchaeota are the two major phyla of archaea, present in high abundance in diverse habitats such as marine (DeLong, 1992, 1998; Orphan et al., 2001), freshwater (Auguet et al., 2010), and soil systems (Ke et al., 2014). The majority of archaea contain membrane lipids known as isoprenoid glycerol dibiphytanyl glycerol tetraethers (iGDGTs) (De Rosa and Gambacorta, 1988). The iGDGTs have the 2,3-di-*O*-alkyl-*sn*-glycerol backbone that is ether linked to dibiphytane chains with zero to as many as eight cyclopentyl rings (see structures in **Supplementary Figure S1**) (Uda et al., 2004; Schouten et al., 2007). The intact polar lipids (IP-iGDGTs) containing sugar, phosphate or both head groups attached to the core lipids of iGDGTs (C-iGDGTs) have been found to degrade rapidly with cleavage of polar head groups after cell death (White et al., 1979; Harvey et al., 1986). They are commonly used as indicators for living microbes (Lipp et al., 2008; Lincoln et al., 2014), despite the possibility that glycolipids can be preserved over a geological timescale (Lengger et al., 2013, 2014). The C-iGDGTs, on the other hand, can be preserved in sediments over millions of years and have been widely used in paleoenvironment studies (Kuypers et al., 2001).

The number of cyclopentyl rings was observed to increase with temperature in pure cultures (Uda et al., 2001, 2004). Subsequently, based on the investigation of global marine surface sediments, Schouten et al. (2002) established a proxy TEX_86_ (tetraether index of tetraethers with 86 carbons), which correlated linearly with sea surface temperature (SST). The TEX_86_ proxy was then applied to reconstruct paleotemperature in marine system (Schouten et al., 2002; Kim et al., 2010) and extended in lacustrine water (Powers et al., 2010; Tierney et al., 2010). This paleotemperature proxy relies on the distribution of iGDGTs-1, -2 and -3, and crenarchaeol isomer.

While TEX_86_ concerns with the relatively low abundances of iGDGTs-1, -2 and -3 and crenarchaeol isomer, crenarchaeol is commonly the most abundant iGDGT identified in pure cultures of ammonia-oxidizing Thaumarchaeota (Schouten et al., 2013). It has been observed to have a good correspondence with thaumarchaeotal *amoA* gene abundance in natural environments (Leininger et al., 2006; De la Torre et al., 2008; Li et al., 2013). Therefore, the predominance of crenarchaeol in the iGDGT profiles of normal marine surface sediments has allowed scientists to believe that marine iGDGTs forming TEX_86_ are mostly produced by Thaumarchaeota (Schouten et al., 2002; Powers et al., 2004; Blaga et al., 2009). However, Marine Group I Thaumarchaeota are found to be predominant in subsurface and deeper ocean (DeLong et al., 1999) while TEX_86_-relevant iGDGTs record the SST signal. Recently, Marine Group II Euryarchaeota were proposed as a significant source of these iGDGTs in the ocean using the integrated analysis of archaeal lipids and community DNA structure (Lincoln et al., 2014), which open up the possibility that crenarchaeol may not be solely produced by Thaumarchaoeta. Testing of this hypothesis has evolutionary implications as fossil crenarchaeol in geological record has been used to indicate the functional radiation of ammonia oxidation from geothermal systems into low temperature environment (Gubry-Rangin et al., 2015).

In a similar way, TEX_86_ was reported to record temperature change along altitudinal transects in terrestrial settings (Liu et al., 2013; Coffinet et al., 2014). Crenarchaeol and its isomer have been shown to be distributed in global soils (Weijers et al., 2006), which harbor distinct ecosystems dominated by different archaeal communities (Hu et al., 2013; Mach et al., 2015). Therefore, the different soil ecosystems may provide an opportunity to examine contrasting associations of the crenarchaeol production.

**FIGURE 1:**
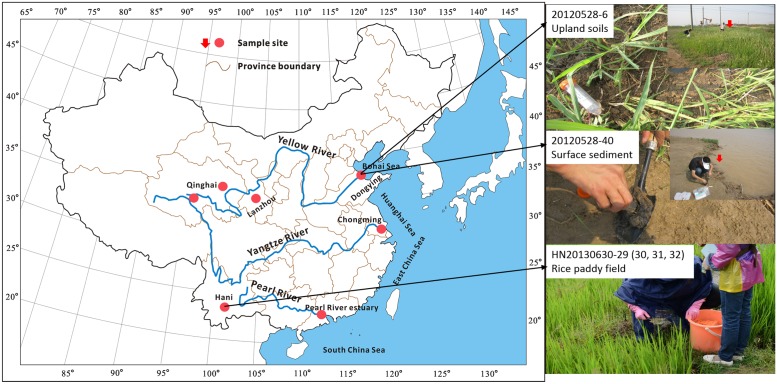
Geographic map of spatial distribution of sampling locations in China.

In this study, 47 surface soils were collected from upland and wetland soils and rice fields; and another three surface sediments were collected from river banks (**Figure 1**). Core structures of IP-iGDGTs and C-iGDGTs, monoglycosidic-iGDGTs (1G-iGDGTs) and diglycosidic-iGDGTs (2G-iGDGTs) were measured to characterize the iGDGT distributions in different fractions. Archaeol in core lipids (C-archaeol, see structure in **Supplementary Figure S1**) was determined to decipher its relationship with the C-iGDGTs. Geographic locations (i.e. latitude, longitude, and elevation) and environmental factors (i.e., temperature, pH, soil water content, ammonium, nitrite, nitrate, TOC, TC, TN) were measured to evaluate the difference in the iGDGT distributions along these gradients. Pyrosequencing and quantification of archaeal 16S rRNA gene enabled us to correlate iGDGT distribution with archaeal community DNA composition. Our study indicates that uncultured representatives of Thermoplasmatales and archaeol-producing methanogens may be associated with production of crenarchaeol in soils in China dominated by these archaea.

## Materials and Methods

### Sample Collection and Environmental Parameter Determination

50 samples were collected from different ecosystems including upland and wetland soils, rice fields and river bank sediments (See **Figure 1** and Supplementary Table S1). These samples were distributed in six geographical locations of China: Dongying (DY), Lanzhou (LZ), Chomgming (CM), Hani (HN), Pearl River estuary (PR), and Qinghai (QH) (See **Figure 1** and Supplementary Table S1). PR samples were collected in January and June 2012, labeled with prefixes PR1201 and PR1206, respectively; DY samples were collected in May 2012; CM samples were collected in July 2012; QH samples were collected in July 2012, labeled with prefixes QHS and BHS; another QH sample was collected in August 2012, labeled with 20120814-15; LZ samples were collected in August 2012; HN samples were collected in June 2013. Replicate samples were not collected at each sampling location. Among these samples, surface samples were collected with the interval between 5 and 15 cm depth from the upland soils and the rice non-flooded field, and with the interval between 0 and 5 cm depth from the rice flooded paddy field and the sediments of river bank (See the detailed description in Supplementary Table S1). All samples were stored at –80°C for further analysis.

Multiple environmental parameters were determined in the current study. The annual mean air temperatures (MAT) were obtained from the climate stations nearest the sampling sites and represented a 30 year average over the period 1981–2010. According to the method described in Weijers et al. (2007), the MAT values were corrected in Lanzhou, Hani and Qinghai using a linear regression model for the relationship between temperature and altitude from two nearby climate stations. The measurement of pH and soil water content (SWC) was performed by mixing a sample with deionized water in the ratio 1:2.5 and by drying samples overnight at 105°C, respectively. The extraction of ammonium, nitrite and nitrate was conducted using 2 M KCl in a ratio of 1:10 (Maynard and Kalra, 1993) and then the extracts were filtered using 0.45 μm pore-size membrane filters. The indophenol blue spectrophotometric method was used to analyze ammonium (Pai et al., 2001), and nitrite and nitrate were examined using a Technicon AA3 Auto-Analyzer (Bran-Lube). Total organic carbon (TOC), total carbon (TC), and total nitrogen (TN) were determined using an organic element analyzer (Carlo-Erba model EA1110, Italy) with TOC samples washed by 1 M HCl to remove any carbonates.

### Lipid Extraction and HPLC-MS Analysis

The total lipids were extracted using a modified Bligh-Dyer method with a solvent mixture of MeOH/DCM/phosphate buffer at pH 7.4 (2:1:0.8, v:v:v) for four times as procedures described in Tierney et al. (2012). Then, the total Bligh-Dyer Extracts (BDEs) were divided into three aliquots. An aliquot of each sample was directly analyzed as C-iGDGTs in the atmospheric pressure chemical ionization (APCI) mode on high-performance liquid chromatography-mass spectrometry (HPLC-MS) (1200 Series/6460 Triple Quad, Agilent Technologies, Santa Clara, CA, United States). The second aliquot was subject to acid hydrolysis (Li et al., 2013) and then detected in HPLC/APCI-MS. The HPLC/APCI-MS was set following the conditions published in Li et al. (2013). The IP-iGDGTs were calculated using subtraction method between non-hydrolyzed and hydrolyzed fractions (Li et al., 2013). The iGDGT-4 in the C- and IP-iGDGT fractions was corrected by subtraction of the signal of crenarchaeol isotope using method as described in Li et al. (2013).

The last aliquot was analyzed in the electrospray ionization (ESI) mode on HPLC/MS following procedures in Chen et al. (2016). Crenarchaeol isomer and iGDGT-4 co-eluted and the contribution from the isotope of crenarchaeol isomer to the signal of iGDGT-4 has not been evaluated in this study. Therefore, iGDGT-4 was not analyzed in the fractions of 1G- and 2G-iGDGTs. The absolute quantification of 1G and 2G-iGDGTs couldn’t be achieved due to a lack of authentic internal standards. Instead, the relative concentrations were reported in this study.

### DNA Extraction and 454 Pyrosequencing

DNA was extracted from about 0.5 g of each sample using the FastDNA SPIN Kit for soil (MP Biomedicals, Santa Ana, California, USA), following the manufacture’s protocol. The 454 pyrosequencing analysis was performed as described in Liu J. et al. (2014). The V3, V4, and V5 regions of the archaeal 16S rRNA gene were targeted using the primers 344f and 915r (Casamayor et al., 2002). The fragments were amplified using a barcoded forward primer and a reverse primer. The PCR program (using TransStart Fastpfu DNA polymerase) was set in ABI GeneAmp^TM^ PCR System 9700 as follows: 95°C for 2 min; 30 cycles of 30 s at 95°C, 30 s at 55°C, and 30 s at 72°C; followed by 5 min at 72°C. Amplicons were pooled, purified and quantified. The purified DNA products were subject to sequencing using Roche GS FLX+ instrument with the Roche GS FLX Titanium Sequencing Kit XLR70. The raw sequence reads have been deposited in the European Nucleotide Archive (ENA) database under the study project accession number PRJEB20534 (sample accession number: ERS1681927-ERS1681976). The pyrosequencing data has been submitted to Qiita under the study number 11167^1^.

The qPCR assays on the archaeal 16S rRNA gene used the same primers 344f/915r. The annealing and extension temperatures for PCR and qPCR programs generally followed the PCR condition as described in the pyrosequencing analysis (See Supplementary Methods). Quantification standard was derived from a positive clone and comprised a dilution series. Each sample and standard contained triplicate measurements. For each run, the *R*^2^ value for standard curve was more than 0.99 and the corresponding efficiency was more than 90%.

### Processing of Pyrosequencing Data

We used QIIME (Caporaso et al., 2010) to process the 454 pyrosequencing data on archaeal 16S rRNA gene. The 454 reads were filtered and low quality reads were excluded using the following quality threshold parameters: the minimum sequence length = 300, the minimum average quality score = 25, the maximum number of ambiguous = 6, the maximum length of homopolymer run = 6. All multiplexed reads were assigned to samples according to the sample-specific barcoded sequences and then primers and barcodes were removed for downstream analyses. All chimeric sequences were detected and removed using USEARCH based on the reference sequences (SILVA release 115). All non-chimeric sequences were clustered into Operational Taxonomic Units (OTUs) at a 99% similarity level. A representative sequence was extracted from each OTU by choosing the most abundant sequence, and then its taxonomy was assigned by comparing with the reference sequences (SILVA release 115) using uclust.

### Statistical Analysis

Cluster analysis on the iGDGT distribution was performed in R using the method described in Li et al. (2013). Briefly, the Euclidean measurement was used to calculate the distance matrix and the complete method was used to establish a hierarchical clustering tree.

The iGDGTs were projected on a phylogenetic tree of Archaea based on 16S rRNA gene, showing difference in the iGDGT composition between archaeal orders (Pearson and Ingalls, 2013). These authors suggested a potential linkage between the iGDGT distribution and archaeal phylogeny. In our study, we selected the composition of archaeal orders to explain the iGDGT distribution. Any orders detected in no more than five samples were removed from the further analysis. As a result, 21 archaeal orders were retained and normalized. These selected organisms contributed more than 94% (usually more than 99%, data not shown) to the archaeal populations in each sample.

The relationship between iGDGTs and archaeal orders, geographic and environmental factors was assessed with the DistLM routine in PRIMER V6.1.16 & PERMANOVA+ V1.0.6 using a step-wise selection procedure and *R*^2^ for selection criterion. The individual iGDGTs in each lipid fraction were imported as the response variables. The resemblance matrix for iGDGT distribution between samples was calculated by the Euclidean method. The individual archaeal organisms and geographic and environmental factors were set as the predictor variables. To account for skewed distributions, ammonium, nitrite, nitrate, TOC, TC, and TN data were log transformed. The conditional test was performed to examine the proportion of each predictor variables conditional upon variables which have been selected to be included in the model. *P* values were obtained using the 999 permutation test on residuals under the reduced model. Results were visualized with a distance based redundancy analysis (dbRDA) on the first two axes, which explained the majority of the bGDGT distribution. We used the criterion of *P*-value <0.05 for selecting the significant predictor variables. Only the significant predictor variables were shown on the dbRDA plot.

## Results

### Distribution of iGDGTs in IP-, C-, 1G-, and 2G-iGDGT Fractions

The concentration of total IP-iGDGTs varied from 0.56 ng/g dry soil (CM12723-35) to 344.75 ng/g dry soil (gds) (HN20130630-30) and that of the total C-iGDGTs between 1.37 ng/gds (CM12723-29) and 185.04 ng/gds (HN20130630-31) (Supplementary Table S1). The 1G- and 2G-iGDGT fractions were identified in ESI mode, which could only give relative abundance in peak areas.

**FIGURE 2:**
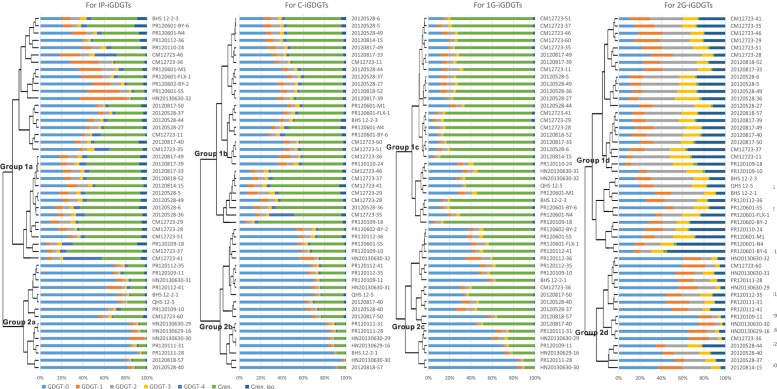
Cluster analysis on the IP-, C-, 1G-, and 2G-iGDGT distributions.

Cluster analysis was performed to identify the difference between the patterns of the iGDGT distributions in the IP-, C-, 1G-, and 2G-iGDGT fractions (**Figure 2**). Generally, the IP-iGDGTs had similar distribution as the C-iGDGTs (**Figure 2**). The 1G-iGDGT distribution had smaller dissimilarity than the 2G-iGDGT distribution to that of the IP-iGDGT fraction (**Figure 2**). Analysis of Similarity (ANOSIM) and Similarity Percentage (SIMPER) generally yielded similar results of iGDGT distribution in different fractions (Supplementary Table S2).

In each iGDGT fraction, these soil samples were clustered into two major groups (**Figure 2**). On average, Group 2 type samples were characterized by greater abundance of iGDGT-0 whereas Group 1 type samples contained greater abundance of crenarchaeol, crenarchaeol isomer, and iGDGTs-2 and -3.

### The DistLM Analysis

**FIGURE 3:**
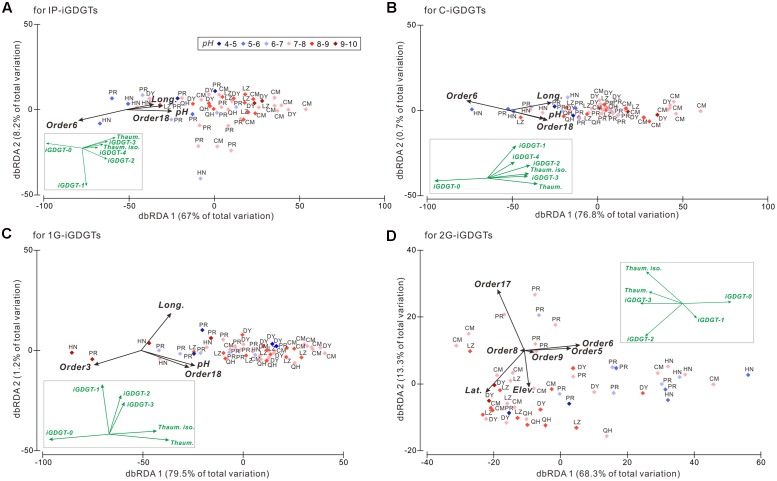
The distance based redundancy analysis (dbRDA) plot showing the isoprenoid glycerol dibiphytanyl glycerol tetraether (iGDGT) distributions against the geographic and environmental parameters and the archaeal community at the order level among samples in the IP- **(A)**, C- **(B)**, 1G- **(C)**, and 2G-iGDGT **(D)** fractions. The black vectors represent predictor variables including the geographic and environmental parameters and the archaeal community DNA composition. Only the significant predictor variables (*P* < 0.05) are shown in the plot. The iGDGT patterns are shown as the green vectors and inserted in each panel for comparison with the predictor variables. Order3, Methanobacteriales; Order5, Methanocellales; Order6, Methanomicrobiales; Order8, Thermoplasmatales; Order9, Undefined order in Thaumarchaeota; Order17, Group I.1b A; Order18, Group I.1b B. Both Order17 and 18 were assigned to Soil Crenarchaeotic Group (SCG) in the processing of the 454 tag sequences using SILVA 115 database. Soil Crenarchaeotic Group is also known as Group I.1b and thus we named Order17 as Group I.1b A and Order18 as Group I.1b B.

The DistLM analysis was performed to identify the relationships between the iGDGTs and predictor variables including the 21 archaeal orders and geographical and environmental factors. The results were shown on the dbRDA plots (**Figure 3**). The biological, geographical and environmental variables collectively explained 67–79.5% of the iGDGT distribution on the axis 1 and 0.7–13.3% on the axis 2 (**Figure 3**). As a result, the axis 1 was focused in the downstream analysis. Furthermore, iGDGT-1 in IP-iGDGTs and iGDGTs-1 to 3 in 1G-iGDGTs loaded much less on the axis 1 (**Figure 3**), indicating that only a very small proportion of these iGDGTs was expressed by axis 1. Therefore, these iGDGTs weren’t included in the relationship analysis between iGDGTs and significant predictor variables. These selection steps minimized the bias caused by the introduction of iGDGTs in which only a small proportion was explained by predictor variables.

The significant predictor variables with *P* < 0.05 were shown in **Table 1** for deciphering the proportion of the iGDGT distribution explained by each of these variables and the cumulative proportion. For example, Methanomicrobiales (Order6) alone explained 31% of variance in the IP-iGDGT distribution whereas Group I.1b B (Order18) conditional upon Methanomicrobiales (Order6) explained 7% in the model and the cumulative proportion from these two variables was 38% (**Table 1**). Generally, Methanomicrobiales (Order6) among the predictor variables explained the greatest proportion of the variance in each iGDGT fractions, except 1G-iGDGTs that were explained largely by pH instead (**Table 1**). Additionally, Thermoplasmatales (Order8) as the significant variable explained 3% of the variance in the 2G-iGDGTs, which was slightly more than ammonium did (*P* > 0.05, Prop. = 2%) (**Table 1**).

**Table 1:** The conditional test of the isoprenoid glycerol dibiphytanyl glycerol tetraether (iGDGT) distributions against environmental factors and archaeal communities at the order level based on the DistLM routine.

	Variable	*P*	Prop.	Cumulative
IP-iGDGTs	Order6	0.001	0.31	0.31
	Order18	0.016	0.07	0.38
	pH	0.031	0.05	0.43
	Long.	0.019	0.05	0.48
C-iGDGTs	Order6	0.001	0.29	0.29
	Long.	0.001	0.14	0.43
	Order18	0.005	0.09	0.52
	pH	0.017	0.06	0.58
1G-iGDGTs	pH	0.001	0.34	0.34
	Order18	0.003	0.13	0.47
	Long.	0.008	0.07	0.54
	Order3	0.004	0.07	0.61
2G-iGDGTs	Order6	0.001	0.31	0.31
	Order17	0.004	0.10	0.41
	Order5	0.01	0.08	0.49
	Lat.	0.019	0.05	0.54
	Order9	0.031	0.05	0.58
	Elev.	0.024	0.04	0.62
	NH4^+^	0.128	0.02	0.64
	Order8	0.049	0.03	0.67

*The significant variables were shown with *P* < 0.05, except ammonium (*P* > 0.05), which was selected into the model prior to Order8 in the DistLM analysis. The order number was referred to the taxonomy name in **Figure 3**.*

Samples with characteristic environments were distributed on the dbRDA plot based on the compositions of iGDGTs. For example, along the axis 1 of dbRDA plot, the left panel (or left side) samples with low longitude and low pH contained more abundant iGDGT-0 whereas the right panel (or right side) samples with high longitude and high pH were characterized by greater abundance of creanrchaeol, crenarchaeol isomer and iGDGTs-2 and -3 in the IP-, C- and 1G-iGDGTs, which is consistent with the results of cluster analysis (**Figure 2**).

On the axis 1 of dbRDA plot, longitude, pH and Group I.1b B (Order18) correlated positively with highly methylated iGDGTs (i.e., iGDGTs-1 to 4, crenarchaeol and its isomer), and negatively with less methylated iGDGT-0 in the IP-, C-, and 1G-iGDGTs (**Figures 3A–C**). Meanwhile, Methanomicrobiales (Order6) in the IP- and C-iGDGTs and Methanobacteriales (Order3) in the 1G-iGDGTs correlated positively with iGDGT-0 and negatively with highly methylated iGDGTs (**Figures 3A–C**). In the 2G-iGDGT fraction, latitude, Group I.1b A (Order17) and Thermoplasmatales (Order8) correlated positively with iGDGTs-2 to 4, crenarchaeol and its isomer and negatively with iGDGTs-0 and 1, whereas the opposite correlations were present in elevation, Methanocellales (Order5), Methanomicrobiales (Order6) and Order9 (unidentified order in Thaumarchaeota) (**Figure 3D**). Additionally, Thermoplasmatales (Order8), Order9 and elevation had very small loading on axis 1 (**Figure 3D**), indicating that these variables had only minor contribution to changes in the 2G-iGDGT distribution.

### Compositions of Archaeal Community and Relationship to Crenarchaeol

Pyrosequencing of the 16S rRNA gene of archaea allowed us to identify candidate populations responsible for the occurrence of crenarchaeol in soils and surface sediments of river bank in China. Among the 50 samples, a total of 119,440 high-quality archaeal 16S rRNA gene sequences were obtained from the barcoded pyrosequencing (Supplementary Table S3). The coverage ranged from 802 to 6,803 sequences per sample with an average of 2,389 reads per sample (Supplementary Table S3). The OTU richness per sample varied between 116 and 906 with an average of 427 at the 99% identity level (Supplementary Table S3). Although the full extent of archaeal diversity was not surveyed (i.e., absence of clear asymptotes in the rarefaction curves; data not shown), this depth of sequencing can accurately capture the dominant lineages and infer the relative abundances of overall taxon (Fierer et al., 2008; Lauber et al., 2009; Bates et al., 2011).

The majority of the archaeal phylotypes were affiliated within Euryarchaeota (34,995 sequences, 29.3% of total archaeal sequences) and Thaumarchaeota (84,418 sequences, 70.7% of total archaeal sequences). Of these Euryarchaeota sequences, 17,127 (48.94% of total euryarchaeotal sequences) were affiliated within methanogens (including orders Methanobacteriales, Methanocellales, Methanomicrobiales, and Methanosarcinales) and 11,812 (33.8% of total euryarchaeotal sequences) represented lineages from the order Thermoplasmatales (Supplementary Table S3). Within the methanogens is the newly identified order Methanoplasmatales, which was previously described as uncultured Thermoplasmatales (Paul et al., 2012). A linkage of Methanomassiliicoccus that belongs to Methanoplasmatales (Paul et al., 2012), however, was assigned to the order Thermoplasmatatles using SILVA release 115 database in this study. It had only nine sequences and thus was not considered as methanogens in our further analysis. In the 84,418 thaumarchaeotal sequences, 71,537 were classified into Group I.1b (including 5,254 from Group I.1b A and 66,283 from Group I.1b B) (Supplementary Table S3). Methanogens and Thermoplasmatales were the two major phylotypes in Euryarchaeota whereas Group I.1b were the dominant Thaumarchaeota.

**FIGURE 4:**
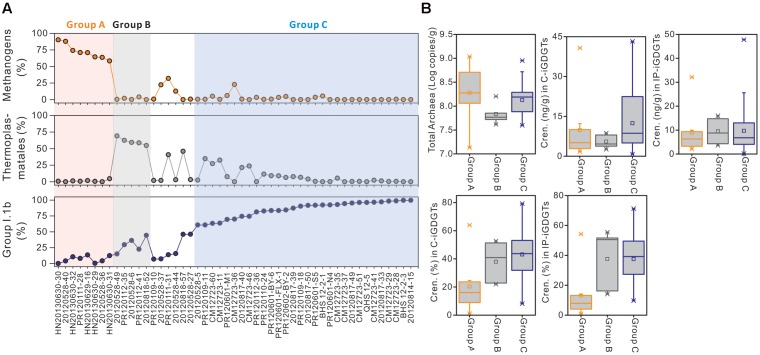
The distributions of methanogens, Thermoplasmatales and Group I.1b Thaumarchaeota among samples **(A)** and comparison of changes in absolute and relative concentrations of crenarchaeol between samples characterized by these three organisms **(B)**. Group A samples labeled with pink color contain more methanogens (50%); Group B samples labeled with gray color contain more Thermoplasmatales (>50%); Group C samples labeled with blue color contain more Group I.1b (>50%). Group A and B samples were in the order in which the relative abundances of the characterized phylotypes (methanogens and Thermoplasmatales, respectively) were ranged from the largest to the smallest, whereas Group C samples were in the order in which Group I.1b were from the smallest to the largest.

The relative abundances of methanogens, Thermoplasmatales, and Group I.1b Thaumarchaeota all varied widely among different samples from below detection (0.0%) to 90.1, 69.0, and 99.6%, respectively (Supplementary Table S1 and **Figure 4A**). According to the relative abundances of these three phylotypes, 43 of the 49 samples were divided among three groups (Groups A, B and C): Group A had 8 samples that were dominated by methanogens (>50% of total archaeal sequences), Group B had 5 samples that were dominated by Thermoplasmatales (>50% of total archaeal sequences), and Group C had 30 samples that were dominated by Group I.1b (>50% of total archaeal sequences); the remaining 6 samples were not grouped because they contained methanogens, Thermoplasmatales, and Group I.1b that were less than 50% of total archaeal sequences in each group (**Figure 4A**). Sample CM12723-4 was excluded in this analysis, because it was not available for the quantification of IP- and C-crenarchaeol.

The distribution of crenarchaeol and the log-transformed cell abundance of total Archaea were evaluated in all three groups (**Table 2** and **Figure 4B**). The abundance of total Archaea in Group C samples was slightly lower than in Group A samples and a little higher than in Group B samples, whereas the mean values were more similar to each other (**Table 2** and **Figure 4B**), suggesting that the biomass couldn’t be an important effect on the distribution of crenarchaeol. In C-iGDGTs, the absolute concentrations of crenarchaeol were more abundant in Group C samples than in either Group A or B samples, however, sample HN20130630-31 of Group A had much higher abundance of crenarchaeol (40.74 ng/g) than the majority of samples of Group C (**Table 2** and **Figure 4B**). In IP-iGDGTs, the absolute concentrations of crenarchaeol were more similar among the three groups, whereas it was more abundant in sample HN20130630-31 (32.09 ng/g) than most of Group C samples (**Table 2** and **Figure 4B**). In C- and IP-iGDGT fractions, the relative abundances of crenarchaeol in Group C samples were more similar to samples of Group B and more abundant than Group A samples, whereas sample 20120528-36 of Group A contained more abundant crenarchaeol (64.0% in C-iGDGTs and 54.2% in IP-iGDGTs) than the majority of Group C samples (**Table 2** and **Figure 4B**).

**Table 2:** The minimum, maximum and mean values of the total archaea and crenarchaeol in absolute and relative concentrations from different group samples.

	Group A			Group B			Group C		
	Min	Max	Mean	Min	Max	Mean	Min	Max	Mean
Archaeal 16S rRNA gene (log copies/g)	7.13	9.04	8.28	7.62	8.20	7.83	7.60	8.95	8.13
C-cren. (ng/g)	1.70	40.74	9.80	2.63	8.67	5.53	0.79	43.20	12.43
IP-cren. (ng/g)	2.06	32.09	8.99	3.61	16.02	9.49	0.18	47.78	9.67
C-cren. (%)	1.09	64.03	20.31	22.08	52.71	37.98	8.18	79.26	43.15
IP-cren. (%)	0.96	54.21	13.18	14.10	55.48	37.58	9.70	71.24	37.47

*Samples of Groups A, B, and C were defined in **Figure 4**.*

### Bivariate Correlation of Crenarchaeol with Thermoplasmatales and Archaeol

**FIGURE 5:**
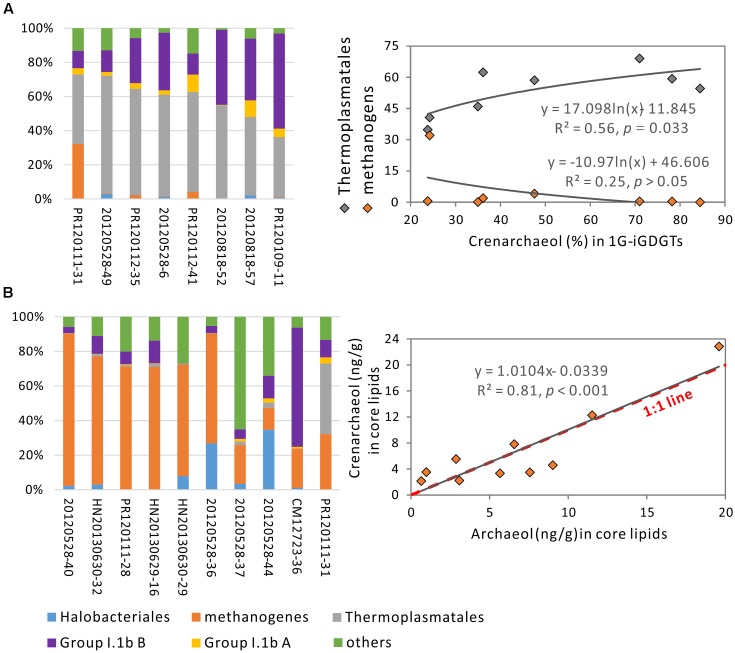
Correlation analysis of crenarchaeol against Thermoplasmatales in eight samples with the presence of this organism of greater than 30% and Group I.1b B less than 60% **(A)** and against archaeol in 10 samples with methanogens of more than 10% **(B)**.

In order to best present the relationships between crenarchaeol and Thermoplasmatales and archaeol, we limited correlation analysis to samples containing abundant Thermoplasmatales or methanogens. According to the distribution of archaeal community, eight samples were composed of enhanced presence of Thermoplasmatales (>30% of total archaea) and reduced Group I.1 B (<60%) (**Figure 5A**). Within these samples, the relative concentration of 1G-crenarchaeol was observed to correlate positively with Thermoplasmatales (*R*^2^ = 0.56, *P* = 0.033) (**Figure 5A**); no significant positive correlation was observed between crenarchaeol and methanogens or archaeol. It must be noted that the relative concentration of Group I.1b B correlated positively with absolute concentration of C-crenarchaeol at the borderline of significance level (*R*^2^ = 0.50, *P* = 0.049, data not shown), indicating that Group I.1b B may still be a source of crenarchaeol in these samples. In the core lipid fraction, iGDGT-2 (*R*^2^ = 0.74, *P* = 0.036) and iGDGT-4 (*R*^2^ = 0.78, *P* = 0.021) in the relative abundance were observed to correlated with Thermoplasmatales (Supplementary Table S5).

Similarly, 10 samples were identified to contain enhanced presence of methanogens (>10% of the total archaea) (**Figure 5B**). In these samples, the absolute concentrations of crenarchaeol correlated positively with the absolute abundance of archaeol in core lipid fraction (*R*^2^ = 0.81, *P* < 0.001) (**Figure 5B**). The slope of the linear regression was very close to that of the 1:1 line, demonstrating that the absolute concentration of crenarchaeol was approximately equal to that of archaeol in these 10 samples. No significant correlations were observed between crenarchaeol and Thermoplasmatales or Group I.1b B. Methanogens were also more than 10% of the total archaeal populations in another two samples HN20130630-30 and -31, which contained C-crenarchaeol (1.70 ng/g) lower than and (40.74 ng/g) higher than that from each of the above 10 samples, respectively (**Supplementary Figure S2**). In the regression model on the correlation between crenarchaeol and archaeol in the core lipids within these 12 samples, the two samples were outliers that had the difference between the predicted and observed values of crenarchaeol equal to (16.61) or higher than (24.10) twice the Root Mean Square Error (RMSE = 8.84) (**Supplementary Figure S2**). After these two samples were removed, the R square was greatly improved from 0.37 to 0.81 and the RMSE reduced from 8.84 to 2.66 (**Figure 5B** and **Supplementary Figure S2**). Therefore, these two samples were considered as influential outliers that were not included in the correlation analysis between crenarchaeol and archaeol in samples with abundant methanogens (**Figure 5B**). Within these 12 samples, the absolute concentration of archaeol also correlated significantly with iGDGT-2 in the intact polar lipid fraction (*R*^2^ = 0.75, *P* = 0.005), and iGDGT-0 (*R*^2^ = 0.60, *P* = 0.039), iGDGT-1 (*R*^2^ = 0.77, *P* = 0.004), and iGDGT-2 (*R*^2^ = 0.70, *P* = 0.011) in the core lipid fraction (Supplementary Table S5).

## Discussion

The iGDGTs are present ubiquitously in nature; however, the studies on their biological sources are primarily limited to pure cultures (Pearson and Ingalls, 2013; Schouten et al., 2013). The majority of archaea thriving in nature are still uncultured and their exact membrane lipids remain essentially unknown. The culture-independent method based on the environmental ribosomal RNA gene analysis has unveiled the wide-spread distribution and much higher diversity of archaea than previously thought (Auguet et al., 2010). Environmental DNA analysis combined with the lipid analysis is a logic approach for constraining the taxonomic origin of the iGDGTs (Schouten et al., 2013; Lincoln et al., 2014). The archaeal taxonomic compositions were hypothesized to connect with the iGDGT distribution (Pearson and Ingalls, 2013). Here, we used the fractional abundance of iGDGTs compared with the relative abundance of archaeal community to explore the association of crenarchaeol production.

Our study reported the wide occurrence of IP-, C-, 1G-, and 2G-iGDGTs in surface soils and riverine surface sediments in China. The similarity of clustering between C- and IP-iGDGTs supports the notion that C-iGDGTs are degradation products of the IP-iGDGTs. Since the IP- and 1G-iGDGTs are much more similarly distributed, it is likely that 1G-iGDGTs account for a much larger portion of the IP-iGDGTs, which is consistent with results from a seawater study (Shiting, 2014). This is supported by the area per g dry weight sample for the total 1G-iGDGTs, which is 2–25 times larger than that for the total 2G-iGDGTs (data not shown). One explanation may be that the 2G-iGDGTs are degraded faster compared with the 1G-iGDGTs (Lengger et al., 2013, 2014).

The organisms producing these iGDGT classes have been identified in pure cultures of Thaumarchaeota, Crenarchaeota, and Euryarchaeota (Pearson and Ingalls, 2013; Schouten et al., 2013), which is supported by our observations from DistLM analysis with all samples considered. For example, Group I.1b A and B correlated positively with iGDGTs-1, 2, 3, 4, crenarchaeol, and its isomer, whereas Methanomicrobiales and Methanobacteriales correlated positively with iGDGT-0. This indicates that these four orders of archaea may have a significant contribution to the lipid pool, in which Methanomicrobiales and Methanobacteriales contribute mostly iGDGT-0 (Sprott et al., 1994, 1999) and Group I.1b, especially Group I.1b B (92.7% of total Group I.1b), contribute the rest of iGDGTs (Pitcher et al., 2010; Sinninghe Damsté et al., 2012). Thermoplasmatales varied positively with iGDGTs-2 and -3, supporting the notion that Thermoplasmatales can synthesize iGDGTs with zero up to eight cyclopentyl rings as observed in pure cultures (Uda et al., 2004).

Among these iGDGTs, crenarchaeol is predominantly associated with Group I.1b Thaumarchaeota (especially Group I.1b B that account for 92.7% of Group I.1b) in the DistLM analysis of our soil samples, supporting that it can be considered as a biomarker for ammonia-oxidizing Thaumarchaeota (Pearson and Ingalls, 2013; Schouten et al., 2013). More interestingly, the relationship of crenarchaeol with Thermoplasmatales in the DistLM model indicate that these organisms may contribute to the change of crenarchaeol pool. Then we presumed that samples dominated by Group I.1b Thaumarchaeota would contain much greater abundance of crenarchaeol than samples dominated by other archaea, if this lipid is only derived from Group I.1b Thaumarchaeota. The similar or higher abundance of crenarchaeol in Group A and B samples (**Figure 4**), compared with that in Group C samples, suggests that the production of crenarchaeol is not exclusively from Group I.1b Thaumarchaeota and may be associated with organisms affiliated within methanogens or Thermoplasmatales.

Our pyrosequencing analysis demonstrated that Group I.1b Thaumarchaeota commonly constitute a large proportion of the archaeal community in upland soils (**Figure 4A** and Supplementary Table S1), which agrees with the previous findings from soils (Bates et al., 2011; Hu et al., 2013). The relationships between crenarchaeol and other archaea may be obscured by the production of crenarchaeol from Group I.1b Thaumarchaeota. Therefore, we performed the correlation analysis only on samples containing large abundance of Thermoplasmatales or methanogens. Thermoplasmatales constituted a major proportion of archaeal populations in eight upland soils (**Figure 5A** and Supplementary Table S1), which is consistent with results from Hu et al. (2013); methanogens were observed in large abundance in 10 samples from rice fields, river surface sediments and wet land (**Figure 5B** and Supplementary Table S1), which are also the case in the previous studies (Hu et al., 2013; Beckmann and Manefield, 2014; Ke et al., 2014; Liu D. et al., 2014; Mach et al., 2015). Additionally, one of the upland soil sites in CM was examined for estimating the monthly changes in archaeal community between March 2012 and March 2013 (Xie et al., 2015). Methanogens, Thermoplasmata, Group I.1b (Nitrososphaera) and YLA114 were dominant in the archaeal populations, which had the maximum dissimilarity in relative abundance less than 6% among different months (Xie et al., 2015). This suggests that the change in archaeal community is not significant among different months, which can be interpreted by that the response of archaeal community DNA structures to environmental change is reduced by their capabilities of the high functional and genetic diversity, rapid evolutionary rate and great dispersal (Prosser et al., 2007; Cruz-Martínez et al., 2009).

When the eight samples with enhanced presence of Thermoplasmatales were considered, the correlation between this group of organisms and crenarchaeol suggests that uncultured representatives of Thermoplasmatales may be mainly responsible for changes in the crenarchaeol pool in these samples.

When the 10 samples containing more abundant methanogens were considered, crenarchaeol was observed in correlation with archaeol in core lipid fraction (**Figure 4A**). Archaeol has been determined mainly from families Methanobacteriaceae, Methanocorpusculaceae, Methanomicrobiaceae, Methanospirillaceae, Methanosaetaceae, and Methanosarcinaceae in methanogens (Koga and Morii, 2005) and Halobacteriaceae in Halobacteriales (Koga and Morii, 2005), which were observed to inhabit these 10 samples. The pure culture *Nitrosopumilus maritimus* of Marine Group I Thaumarchaeota has been detected to produce archaeol as well (Elling et al., 2016), however, only two sequences were from *Nitrosopumilus*-like organisms that were present in sample 20120528-36 of all 50 soil samples. Within these 10 samples, 9,016 sequences were from archaeol-producing methanogens whereas 62 sequences were from Halobacteriaceae (Supplementary Table S4). Thus, archaeol may be predominantly derived from methanogens in these 10 samples, consistent with previous findings in water-saturated soils (Lim et al., 2012) and peatlands (Pancost et al., 2011). However, no significant positive correlations were observed between relative concentration or OTU richness of methanogens and crenarchaeol, which suggests that crenarchaeol may be synthesized by uncultured representatives of methanogens that could produce archaeol. Furthermore, the generally equal values of crenarchaeol and archaeol in these 10 samples suggest that creanarchaeol may be as important as archaeol in the membrane lipids of uncultured representatives of methanogens. Another two influential outliers HN20130630-30 and -31 have crenarchaeol much less important and much more important than archaeol, respectively (**Supplementary Figure S2**).

When the cutoff value was decreased and more samples were added in the bivariate correlation analysis, the *R*^2^ value couldn’t be enhanced. For example, for methanogens, when the cutoff value was reduced to 6%, *R*^2^ was reduced to 0.79; when the cutoff value was reduced to 5%, *R*^2^ was reduced to 0.11. For Thermoplasmatales, when adding one sample CM12723-11 [containing Thermoplasmatales (32.48%) and Group I.1b B (60.20%)], *R*^2^ was reduced to 0.22; when adding two samples CM12723-11 and CM12723-60 [containing Thermoplasmatales (27.34%) and Group I.1b B (59.90%)], *R*^2^ was reduced to 0.06. One explanation is that the crenarchaeol production may be mainly associated with Thermoplasmatales and methanogens in the samples selected in our current study and associated with other organisms such as Group I.1b Thaumarchaeota beyond these samples.

Among all 50 samples, eight samples were used for the correlation analysis between Thermoplasmatales and crenarchaeol and 10 samples for the correlation analysis between archaeol and crenarchaeol when 30 samples characterized by Group I.1 b Thaumarchaeota were excluded. Although the number of samples was not balanced, these limited samples characterized by Thermoplasmatales and methanogens provided an opportunity to show that the crenarchaeol producer might be affiliated with non-thaumarchaeotal archaea. Furthermore, within these samples, the two euryarchaeotal organisms were identified to correlate with iGDGTs-0, -1, and -2, which is overall consistent with the results of DistLM analysis. The iGDGT production from pure cultures of Thermoplasmatales and methanogens (Schouten et al., 2013) suggests that this bivariate correlation method is reliable for identifying the association of iGDGT producers.

This study suggests that methanogens and Thermoplasmatales may be associated with production of these two lipids. One explanation is that crenarchaeol and its isomer were synthesized by representatives of these two micro-organisms in the euryarchaeotal phylum, which is consistent with the proposal of Marine Group II being the source of crenarchaeol (Lincoln et al., 2014). The other explanation is that these two groups of microorganisms have systematic networks with the producers of crenarchaeol. Nevertheless, these results implicate that crenarchaeol may be more commonly present in the domain Archaea than previously recognized. So far, crenarchaeol has been considered as the biomarker for ammonia-oxidizing Thaumarchaeota (Schouten et al., 2013). Its cyclohexyl moiety was hypothesized to prevent the dense packing of tetraethers, which enables the source organisms to thrive in low temperature environments (Sinninghe Damsté et al., 2002). Later, crenarchaeol was found in hot springs (Pearson et al., 2004; Zhang et al., 2006), which implied that crenarchaeol might be an ancient trait of archaea that allowed the transition of thermophilic species to the low temperature environment. Our study suggests that the ecological function of crenarchaeol may be impacted by more factors other than those from Thaumarchaeota. Also, the underlying mechanism of GDGT variation in modern and ancient environments may be complicated by the role of Thermoplasmatales and archaeol-producing methanogens. However, crenarchaeol hasn’t been reported in any isolates of these two orders. In the future, enrichments, pure cultures and isotopically labeled tracer experiments in organisms of these two orders are all needed to better constrain the taxonomic origins of crenarchaeol and understand the ecological and physiological functions of these organisms.

## Author Contributions

FL and CZ designed the work, analyzed data, and wrote the manuscript; FL and FZ conducted experiment; all authors contributed to the collection of samples.

## Conflict of Interest Statement

The authors declare that the research was conducted in the absence of any commercial or financial relationships that could be construed as a potential conflict of interest.

## Footnotes

1 https://qiita.ucsd.edu/

## Supplementary Material

FIGURE S1 Structures of polar head groups **(A)** and core iGDGTs and archaeol **(B)** examined in this study. Cren., Crenarchaeol; Cren. iso., Crenarchaeol isomer.

FIGURE S2 Correlation analysis of crenarchaeol against archaeol in 12 samples with methanogens more than 10%.
