# Supplementary figures and images for: Thermoplasmatales and Methanogens: Potential Association with the Crenarchaeol Production in Chinese Soils
Source: Front Microbiol. 2017 Jun 30;8:1200. doi: 10.3389/fmicb.2017.01200 (PMC5494375; doi:10.3389/fmicb.2017.01200)

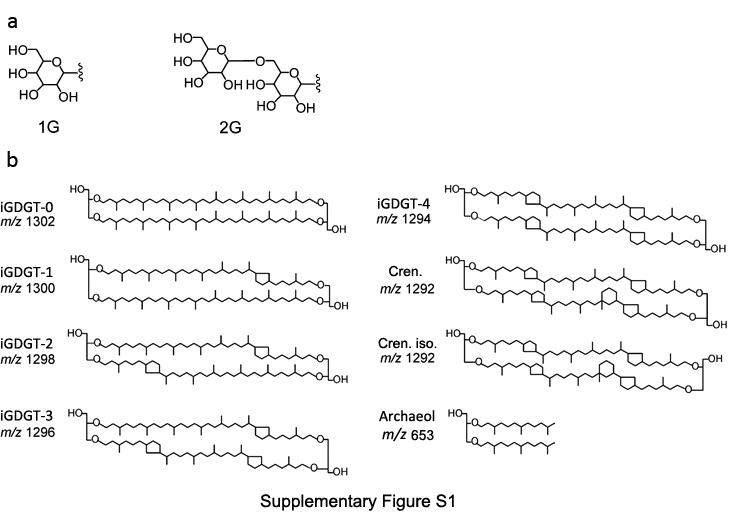

Supplement: FIGURE S1 — Structures of polar head groups (A) and core iGDGTs and archaeol (B) examined in this study. Cren., Crenarchaeol; Cren. iso., Crenarchaeol isomer. [file Image_1.TIF]

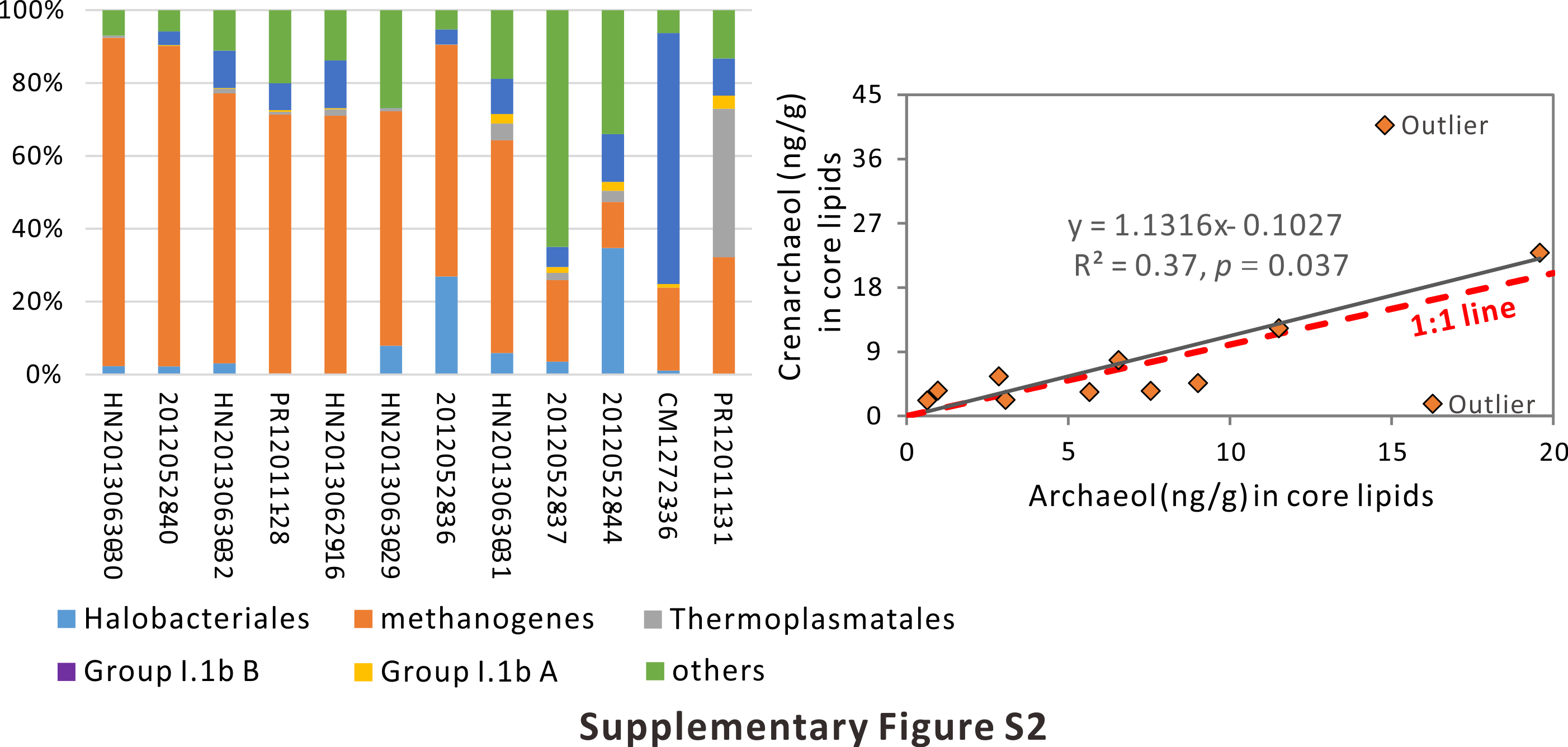

Supplement: FIGURE S2 — Correlation analysis of crenarchaeol against archaeol in 12 samples with methanogens more than 10%. [file Image_2.TIF]
