# Supplementary material for: Thermoplasmatales and Methanogens: Potential Association with the Crenarchaeol Production in Chinese Soils
Source: Front Microbiol. 2017 Jun 30;8:1200. doi: 10.3389/fmicb.2017.01200 (PMC5494375; doi:10.3389/fmicb.2017.01200)
Supplement: Supplementary file 3 [file Data_Sheet_1.DOCX]

Supplementary Methods

PCR and qPCR analysis

PCR with Takara Ex Taq was run on an Eppendorf Mastercycler Ep Gradient S thermocycler as follows: an initial denaturation of 1 minute at 94 °C; 30 cycles of 30 seconds at 94 °C, 30 seconds at 55 °C, and 30 seconds at 72 °C; and a final 5 minute elongation at 72 °C.

The qPCR reactions with SYBR Premix Ex Taq II were performed on Thermo PIKOREAL 96 Real-Time PCR system as follows: an initial denaturation of 30 seconds at 95 °C; 30 cycles of 15 seconds at 95 °C, 30 seconds at 55 °C, and 30 seconds at 72 °C; and a final 10 minute elongation at 72 °C.
