# Supplementary material for: Thermoplasmatales and Methanogens: Potential Association with the Crenarchaeol Production in Chinese Soils
Source: Front Microbiol. 2017 Jun 30;8:1200. doi: 10.3389/fmicb.2017.01200 (PMC5494375; doi:10.3389/fmicb.2017.01200)
Supplement: Supplementary file 4 [file Table_1.DOCX]

Supplementary Table S1. Sample information on geographic (location, latitude (Lat.), longitude (long.), elevation (Elev.)) and environmental (MAT, pH, SWC, ammonium, nitrite, nitrate, TOC, TC, TN) parameters, total iGDGT concentrations and relative abundances of the selected archaeal phylotypes at the order level discussed in the main text.

|  |  |  | Lat. | Long. | Elev. | MAT |  | SWC | NH_4_^+^ | NO_2_^-^ | NO_3_^-^ | TOC | TC | TN | Total concentrations (ng/g) | | |  | Selected phylotypes (%) | | | | | | |
| --- | --- | --- | --- | --- | --- | --- | --- | --- | --- | --- | --- | --- | --- | --- | --- | --- | --- | --- | --- | --- | --- | --- | --- | --- | --- |
| Sample | Location | Description | (°) | (°) | (m) | (°C) | pH | (%) | (umol/L) | (umol/L) | (umol/L) | (%) | (%) | (%) | IP-iGDGTs | C-iGDGTs | Archaeol  in core lipids |  | Methanob-acteriales | Methano-cellales | Methanom-icrobiales | Methanos-arcinales | Thermopla-smatales | Group I.1b A | Group I.1b B |
| 20120528-27 | DY | Surface soil in grassland | 37.81 | 118.80 | 5 | 12.60 | 7.66 | 16.40 | 32.95 | 0.21 | 185.22 | 0.25 | 3.02 | 0.14 | 58.67 | 55.99 | 17.52 |  | 0.00 | 0.00 | 0.19 | 0.56 | 3.18 | 0.00 | 46.07 |
| 20120528-36 | DY | Surface soil in wetland close to a river | 37.78 | 119.08 | -6 | 12.60 | 9.22 | 22.64 | 20.21 | 0.21 | 7.91 | 0.65 | 1.93 | 0.07 | 11.71 | 19.16 | 11.52 |  | 0.00 | 0.00 | 0.00 | 63.61 | 0.12 | 0.00 | 4.10 |
| 20120528-37 | DY | Surface sediment on the bank of a river | 37.78 | 119.08 | -5 | 12.60 | 8.13 | 29.07 | 21.75 | 0.42 | 6.27 | 0.55 | 2.51 | 0.08 | 22.50 | 9.75 | 0.97 |  | 0.13 | 0.04 | 0.81 | 21.39 | 2.00 | 1.62 | 5.50 |
| 20120528-40 | DY | Surface sediment on the bank of a river | 37.76 | 119.12 | -17 | 12.60 | 7.69 | 31.38 | 68.62 | 0.28 | 7.13 | 1.35 | 3.15 | 0.16 | 93.46 | 31.72 | 6.56 |  | 1.40 | 0.74 | 12.64 | 73.12 | 0.03 | 0.24 | 3.75 |
| 20120528-44 | DY | Surface sediment on the bank of a river | 37.76 | 119.09 | -2 | 12.60 | 8.38 | 50.16 | 26.40 | 0.09 | 5.43 | 0.21 | 2.03 | 0.06 | 11.75 | 5.53 | 0.64 |  | 1.35 | 0.03 | 1.98 | 9.33 | 3.03 | 2.43 | 13.11 |
| 20120528-49 | DY | Surface soil in grassland | 37.56 | 118.72 | 5 | 12.60 | 9.24 | 10.64 | 17.31 | 0.11 | 7.51 | 0.23 | 1.39 | 0.06 | 7.15 | 5.12 | 0.52 |  | 0.05 | 0.00 | 0.00 | 0.30 | 69.02 | 2.15 | 12.84 |
| 20120528-5 | DY | Surface soil in grassland | 37.79 | 118.66 | 6 | 12.60 | 7.66 | 12.64 | 41.19 | 0.27 | 606.54 | 0.82 | 2.11 | 0.09 | 28.55 | 17.04 | 1.30 |  | 0.00 | 0.07 | 0.01 | 0.47 | 2.41 | 2.18 | 58.42 |
| 20120528-6 | DY | Surface soil in grassland | 37.79 | 118.66 | 6 | 12.60 | 8.07 | 15.85 | 37.23 | 0.18 | 21.39 | 0.88 | 2.38 | 0.11 | 28.87 | 16.46 | 0.53 |  | 0.00 | 0.20 | 0.00 | 0.10 | 59.29 | 2.56 | 33.73 |
| 20120814-15 | QH | Surface soil in grassland with moss and lichen | 34.91 | 97.53 | 4573 | -4.84 | 7.82 | 31.92 | 272.74 | 0.51 | 12.81 | 4.75 | 5.50 | 0.40 | 105.76 | 48.18 | 1.10 |  | 0.00 | 0.00 | 0.00 | 0.00 | 0.00 | 1.06 | 98.50 |
| 20120817-33 | LZ | Surface soil in grassland in Lanzhou city | 34.07 | 103.85 | 1630 | 8.69 | 8.50 | 14.94 | 51.58 | 13.75 | 71.64 | 2.63 | 2.87 | 0.09 | 22.92 | 10.34 | 0.28 |  | 0.00 | 0.00 | 0.00 | 0.06 | 1.20 | 0.88 | 96.13 |
| 20120817-39 | LZ | Surface soil in a small garden in Lanzhou city | 36.07 | 103.82 | 1630 | 8.69 | 7.91 | 18.13 | 48.56 | 4.52 | 34.47 | 1.71 | 3.35 | 0.15 | 38.27 | 22.73 | 0.52 |  | 0.00 | 0.00 | 0.00 | 0.00 | 7.71 | 9.80 | 77.31 |
| 20120817-40 | LZ | Surface soil in a small garden in Lanzhou city | 36.07 | 103.83 | 1630 | 8.69 | 8.31 | 15.37 | 115.81 | 0.45 | 14.48 | 1.46 | 3.15 | 0.13 | 11.68 | 13.24 | 0.42 |  | 0.10 | 0.00 | 0.03 | 0.57 | 21.45 | 3.16 | 71.14 |
| 20120817-49 | LZ | Surface soil in Baita hill park in Lanzhou city | 36.07 | 103.81 | 1552 | 8.67 | 7.98 | 21.63 | 53.94 | 0.31 | 98.98 | 1.28 | 3.21 | 0.11 | 41.50 | 27.35 | 0.55 |  | 0.00 | 0.00 | 0.00 | 0.02 | 0.36 | 9.00 | 86.19 |
| 20120817-50 | LZ | Surface soil in Lanzhou city | 36.07 | 103.81 | 1530 | 8.56 | 8.15 | 17.74 | 44.04 | 0.21 | 25.57 | 0.78 | 2.44 | 0.08 | 37.15 | 35.78 | 1.85 |  | 0.00 | 0.00 | 0.00 | 0.00 | 4.04 | 16.87 | 74.72 |
| 20120818-52 | LZ | Surface soil in the Yellow River Stone Forest National Geopark | 36.90 | 104.33 | 1336 | 7.58 | 8.32 | 8.78 | 28.85 | 0.28 | 377.92 | 2.27 | 1.31 | 0.06 | 28.63 | 11.23 | 0.41 |  | 0.00 | 0.00 | 0.00 | 0.00 | 54.59 | 0.31 | 43.96 |
| 20120818-57 | LZ | Surface soil in the Yellow River Stone Forest National Geopark | 36.90 | 104.31 | 1336 | 7.58 | 8.64 | 16.15 | 24.96 | 0.16 | 192.93 | 0.70 | 2.22 | 0.09 | 97.33 | 103.02 | 2.54 |  | 0.00 | 0.00 | 0.00 | 0.03 | 45.98 | 9.73 | 36.11 |
| CM12723-11 | CM | Surface soil in a Chinese yam land | 31.50 | 121.81 | 3 | 16.60 | 7.89 | 10.23 | 33.58 | 0.30 | 224.13 | 0.50 | 1.29 | 0.10 | 1.20 | 11.44 | NA |  | 0.09 | 0.00 | 0.34 | 0.00 | 32.48 | 3.40 | 60.20 |
| CM12723-28 | CM | Surface soil in the wood | 31.67 | 121.49 | 2 | 16.60 | 7.74 | 11.77 | 30.34 | 0.85 | 15.53 | 1.67 | 2.16 | 0.19 | 5.25 | 39.02 | NA |  | 0.39 | 0.10 | 0.00 | 0.05 | 0.63 | 0.00 | 98.55 |
| CM12723-29 | CM | Surface soil in the palm | 31.68 | 121.48 | 5 | 16.60 | 8.00 | 20.29 | 92.99 | 0.42 | 28.43 | 2.44 | 3.18 | 0.22 | 6.85 | 1.37 | 0.18 |  | 0.00 | 0.00 | 0.00 | 0.00 | 0.43 | 4.78 | 93.41 |
| CM12723-35 | CM | Surface soil in a corn land | 31.74 | 121.39 | 3 | 16.60 | 7.93 | 21.42 | 65.65 | 1.79 | 31.44 | 1.48 | 1.79 | 0.14 | 0.56 | 10.03 | 0.40 |  | 0.00 | 0.00 | 0.00 | 0.00 | 5.36 | 2.46 | 90.29 |
| CM12723-36 | CM | Surface soil in a rice non-flooded field | 31.75 | 121.39 | 4 | 16.60 | 7.46 | 37.35 | 449.44 | 0.55 | 6.97 | 1.79 | 2.75 | 0.18 | 40.42 | 59.52 | NA |  | 5.99 | 3.78 | 2.50 | 10.47 | 0.03 | 0.93 | 68.93 |
| CM12723-37 | CM | Surface soil in a cotton land | 31.76 | 121.39 | 5 | 16.60 | 7.99 | 20.29 | 57.86 | 0.22 | 73.82 | 1.41 | 2.02 | 0.14 | 10.22 | 13.22 | 0.06 |  | 0.00 | 0.00 | 0.00 | 0.12 | 0.12 | 9.16 | 85.03 |
| CM12723-4 | CM | Surface soil in a taro land | 31.54 | 121.72 | 3 | 16.60 | 7.92 | 20.42 | 62.51 | 0.72 | 9.06 | 1.22 | 1.67 | 0.16 | NA | NA | 19.59 |  | 0 | 0 | 0 | 0 | 3.03 | 9.55 | 81.04 |
| CM12723-41 | CM | Surface soil in a potato land | 31.80 | 121.37 | 6 | 16.60 | 8.06 | 7.56 | 20.50 | 0.28 | 58.42 | 1.07 | 1.82 | 0.13 | 3.22 | 34.82 | 0.14 |  | 0.00 | 0.00 | 0.00 | 0.00 | 2.22 | 0.83 | 95.63 |
| CM12723-46 | CM | Surface soil on the roadside | 31.81 | 121.29 | 4 | 16.60 | 8.52 | 5.30 | 32.10 | 0.93 | 244.54 | 1.26 | 2.07 | 0.12 | 1.25 | 8.57 | NA |  | 0.00 | 0.00 | 0.00 | 0.00 | 23.69 | 4.11 | 70.20 |
| CM12723-51 | CM | Surface soil on the roadside | 31.75 | 121.28 | 3 | 16.60 | 8.30 | 12.38 | 52.02 | 0.27 | 17.23 | 0.64 | 1.46 | 0.07 | 21.73 | 12.81 | NA |  | 0.00 | 0.00 | 0.00 | 0.00 | 0.51 | 0.82 | 95.29 |
| CM12723-60 | CM | Unknown surface soil | 31.58 | 121.55 | 5 | 16.60 | 7.82 | 11.86 | 138.63 | 6.73 | 202.04 | 0.87 | 1.86 | 0.11 | 15.05 | 78.09 | 3.41 |  | 0.61 | 0.78 | 0.09 | 3.56 | 27.34 | 3.30 | 59.90 |
| HN20130629-16 | HN | Surface soil in a rice non-flooded field | 23.11 | 102.74 | 1040 | 20.09 | 7.05 | 23.60 | 16.11 | 0.04 | 6.13 | 0.21 | 1.98 | 0.16 | 44.76 | 22.55 | 2.75 |  | 1.81 | 1.61 | 20.68 | 46.70 | 1.77 | 0.31 | 13.07 |
| HN20130630-29 | HN | Surface soil in a rice paddy field | 23.11 | 102.74 | 1600 | 17.11 | 5.81 | 44.12 | 18.47 | 0.02 | 4.55 | 0.18 | 1.44 | 0.12 | 94.36 | 45.49 | 3.07 |  | 4.05 | 0.61 | 17.07 | 42.65 | 0.57 | 0.12 | 0.12 |
| HN20130630-30 | HN | Surface soil in a rice paddy field | 23.11 | 102.74 | 1600 | 17.11 | 5.95 | 49.63 | 26.66 | 0.10 | 6.07 | 0.15 | 1.03 | 0.08 | 344.75 | 156.54 | 9.02 |  | 7.62 | 0.36 | 18.91 | 63.17 | 0.67 | 0.00 | 0.00 |
| HN20130630-31 | HN | Surface soil in a rice paddy field | 23.11 | 102.74 | 472 | 23.10 | 7.50 | 33.68 | 18.14 | 0.19 | 6.59 | 0.13 | 3.00 | 0.24 | 233.97 | 185.04 | 16.27 |  | 0.79 | 0.25 | 22.17 | 35.15 | 4.62 | 2.61 | 9.64 |
| HN20130630-32 | HN | Surface soil in a rice paddy field | 23.11 | 102.74 | 1250 | 18.97 | 6.98 | 31.65 | 24.11 | 0.24 | 7.13 | 0.40 | 1.61 | 0.24 | 78.90 | 24.33 | 14.79 |  | 0.17 | 0.04 | 11.76 | 62.12 | 1.26 | 0.21 | 10.28 |
| PR120109-10 | PR | NA^a^ | 23.49 | 112.86 | 8 | 22.00 | 4.83 | 26.39 | 18.82 | 0.12 | 35.68 | 1.78 | 1.99 | 0.19 | 83.07 | 49.91 | 2.86 |  | 0.28 | 0.08 | 0.10 | 0.33 | 1.98 | 0.36 | 6.46 |
| PR120109-11 | PR | Upland surface soil | 23.47 | 112.86 | 3 | 22.00 | 6.44 | 13.16 | 32.99 | 0.32 | 89.40 | 0.92 | 2.23 | 0.20 | 37.73 | 38.28 | 10.00 |  | 0.00 | 0.00 | 0.00 | 0.48 | 34.82 | 4.94 | 55.66 |
| PR120109-18 | PR | Upland surface soil | 23.35 | 112.90 | 6 | 22.00 | 6.44 | 11.92 | 24.80 | 0.07 | 18.93 | 1.33 | 1.34 | 0.13 | 21.49 | 14.62 | 1.45 |  | 0.00 | 0.00 | 0.00 | 0.05 | 6.63 | 1.72 | 88.59 |
| PR120110-24 | PR | Upland surface soil | 23.35 | 112.91 | 5 | 22.00 | 6.49 | 12.24 | 17.92 | 0.10 | 5.63 | 0.50 | 0.58 | 0.06 | 8.04 | 3.92 | 0.08 |  | 0.05 | 0.10 | 0.00 | 0.23 | 11.57 | 18.42 | 64.25 |
| PR120111-28 | PR | Surface soil in wetland close to a river | 23.59 | 112.98 | 9 | 22.00 | 5.67 | 25.15 | 17.53 | 0.20 | 59.42 | 0.05 | 1.49 | 0.14 | 93.13 | 43.81 | 0.33 |  | 30.57 | 2.89 | 9.26 | 28.27 | 0.73 | 0.49 | 7.35 |
| PR120111-31 | PR | Upland surface soil | 23.62 | 113.06 | 9 | 22.00 | 6.41 | 14.06 | 18.37 | 0.11 | 5.92 | 1.12 | 1.10 | 0.08 | 58.76 | 38.56 | 7.56 |  | 1.47 | 0.46 | 5.06 | 25.09 | 40.71 | 3.62 | 10.18 |
| PR120112-35 | PR | Upland surface soil | 23.74 | 113.23 | 13 | 22.00 | 6.87 | 17.20 | 18.39 | 0.29 | 28.19 | 1.93 | 1.31 | 0.11 | 54.06 | 34.94 | 5.66 |  | 0.67 | 0.38 | 0.19 | 0.67 | 62.37 | 3.25 | 26.36 |
| PR120112-36 | PR | Upland surface soil | 23.72 | 113.24 | 17 | 22.00 | 4.84 | 18.76 | 71.23 | 0.09 | 42.18 | 2.48 | 2.60 | 0.20 | 14.86 | 5.41 | 2.57 |  | 0.48 | 0.41 | 0.07 | 2.34 | 0.00 | 0.19 | 81.02 |
| PR120112-41 | PR | Upland surface soil l | 23.76 | 113.41 | 21 | 22.00 | 5.52 | 15.87 | 19.84 | 0.05 | 42.60 | 1.24 | 1.21 | 0.11 | 30.42 | 17.08 | 0.63 |  | 0.09 | 0.35 | 0.17 | 3.51 | 58.58 | 10.10 | 12.35 |
| PR120601-BY-6 | PR | Upland surface soil | 23.40 | 112.89 | 8 | 22.00 | 7.58 | 18.58 | 16.89 | 0.31 | 17.07 | 0.97 | 1.09 | 0.09 | 12.45 | 7.36 | 1.83 |  | 0.00 | 0.22 | 0.00 | 0.44 | 9.12 | 16.81 | 66.48 |
| PR120601-FLX-1 | PR | NA | 23.70 | 113.15 | 23 | 22.00 | 7.37 | 22.64 | 19.62 | 0.13 | 10.09 | 0.65 | 1.01 | 0.10 | 106.34 | 102.42 | 1.22 |  | 2.20 | 0.15 | 0.15 | 0.81 | 8.88 | 8.37 | 75.04 |
| PR120601-M1 | PR | NA | 23.70 | 113.04 | 7 | 22.00 | 7.52 | 26.29 | 22.53 | 0.34 | 10.76 | 1.07 | 1.17 | 0.10 | 40.37 | 65.66 | 11.65 |  | 0.12 | 0.15 | 3.36 | 2.52 | 7.65 | 17.18 | 52.14 |
| PR120601-N4 | PR | NA | 23.63 | 112.95 | 6 | 22.00 | 7.18 | 29.71 | 42.99 | 0.40 | 8.43 | 2.40 | 2.54 | 0.23 | 37.79 | 29.78 | 15.45 |  | 0.00 | 0.03 | 0.05 | 0.03 | 0.30 | 9.26 | 83.04 |
| PR120601-S5 | PR | NA | 23.70 | 113.04 | 11 | 22.00 | 7.49 | 27.93 | 29.80 | 0.43 | 7.43 | 1.50 | 1.79 | 0.17 | 63.50 | 94.16 | 3.15 |  | 0.20 | 0.06 | 1.51 | 1.68 | 0.49 | 2.90 | 88.88 |
| PR120602-BY-2 | PR | NA | 23.25 | 112.82 | 8 | 22.00 | 7.18 | 21.39 | 29.76 | 0.11 | 7.59 | 1.01 | 1.01 | 0.07 | 21.61 | 16.70 | 15.96 |  | 0.40 | 0.11 | 1.64 | 2.59 | 6.53 | 10.47 | 73.88 |
| QHS 12-5 | QH | Surface soil in grass land | 36.62 | 100.23 | 3232 | 0.79 | 7.92 | 22.29 | 39.64 | 1.25 | 221.49 | 1.15 | 3.90 | 0.32 | 28.75 | 36.86 | 2.37 |  | 0.00 | 0.00 | 0.00 | 0.00 | 0.00 | 4.74 | 91.41 |
| BHS 12-2-1 | QH | Surface soil in grass land | 37.17 | 99.29 | 3312 | -0.04 | 8.17 | 28.79 | 57.32 | 0.55 | 24.57 | 1.79 | 5.90 | 0.32 | 45.93 | 68.31 | 13.74 |  | 3.90 | 0.10 | 0.13 | 1.35 | 0.03 | 0.88 | 91.25 |
| BHS 12-2-3 | QH | Surface soil in grass land | 37.17 | 99.29 | 3312 | -0.04 | 8.39 | 27.47 | 60.11 | 0.62 | 116.36 | 3.01 | 6.27 | 0.39 | 37.43 | 26.82 | 21.48 |  | 0.00 | 0.00 | 0.00 | 0.00 | 0.00 | 2.06 | 97.46 |

^a^Not available.
