# Supplementary material for: Thermoplasmatales and Methanogens: Potential Association with the Crenarchaeol Production in Chinese Soils
Source: Front Microbiol. 2017 Jun 30;8:1200. doi: 10.3389/fmicb.2017.01200 (PMC5494375; doi:10.3389/fmicb.2017.01200)
Supplement: Supplementary file 5 [file Table_2.DOCX]

Supplementary Table S2. ANOSIM and SIMPER test for comparing the iGDGT distributions between the IP-, C-, 1G- and 2G-iGDGT fractions. The iGDGT species with Average Squared Distance more than 100 is shown.

|  | ANOSIM Test | |  | SIMPER Test | | | | | | |
| --- | --- | --- | --- | --- | --- | --- | --- | --- | --- | --- |
| Fractions | R Statistic | Significance Level % |  | Species | Average Abundance (%) in IP-iGDGT group | Average Abundance (%) in 1G-iGDGT group | Average Abundance (%) in 2G-iGDGT group | Average Squared Distance | Contribution% | Cummulative% |
| IP- and C-iGDGTs | 0.012 | 15.6 |  |  |  |  |  |  |  |  |
|  |  |  |  |  |  |  |  |  |  |  |
| IP-, 1G- and 2G-iGDGTs | 0.526 | 0.01 |  |  |  |  |  |  |  |  |
| IP- and 1G-iGDGTs | 0.173 | 0.01 |  | Cren. | 34.38 | 58.78 |  | 1420 | 49.61 | 49.61 |
|  |  |  |  | GDGT-0 | 42.81 | 28.32 |  | 1300 | 45.64 | 95.25 |
| IP- and 2G-iGDGTs | 0.575 | 0.01 |  | Cren. | 34.38 |  | 1.45 | 1480 | 40.42 | 40.42 |
|  |  |  |  | GDGT-0 | 42.81 |  | 29.64 | 1190 | 32.49 | 72.91 |
|  |  |  |  | GDGT-2 | 6.45 |  | 23.74 | 413 | 11.24 | 84.15 |
|  |  |  |  | Cren. iso. | 4.16 |  | 18.95 | 357 | 9.72 | 93.87 |
|  |  |  |  | GDGT-1 | 8.82 |  | 14.18 | 124 | 3.37 | 97.24 |
|  |  |  |  | GDGT-3 | 3.38 |  | 12.05 | 101 | 2.76 | 100 |
| 1G- and 2G-iGDGTs | 0.832 | 0.01 |  | Cren. |  | 58.78 | 1.45 | 3710 | 65.07 | 65.07 |
|  |  |  |  | GDGT-0 |  | 28.32 | 29.64 | 842 | 14.77 | 79.84 |
|  |  |  |  | GDGT-2 |  | 4.93 | 23.74 | 462 | 8.1 | 87.94 |
|  |  |  |  | Cren. iso. |  | 1.27 | 18.95 | 443 | 7.77 | 95.71 |
|  |  |  |  | GDGT-1 |  | 4.17 | 14.18 | 130 | 2.27 | 97.98 |
|  |  |  |  | GDGT-3 |  | 2.54 | 12.05 | 115 | 2.02 | 100 |

**Method.** The dissimilarity between the IP-, C-, 1G- and 2G-iGDGT fraction patterns was examined using Analysis of Similarity (ANOSIM) and the contribution of the iGDGT species to the dissimilarity was detected using Similarity Percentage (SIMPER) in PRIMER V6.1.16 & PERMANOVA+ V1.0.6. The distance matrix between the four iGDGT fractions was measured using Euclidean method. The pair-wise resemblances were calculated.

**Results.** The ANOSIM and SIMPER tests were employed to evaluate if the dissimilarity between the IP-, C-, 1G- and 2G-iGDGT distributions was significant or not, and to examine the contribution of the iGDGT species to the dissimilarity (Suppl. Table 2). In the ANOSIM test, the R Statistic for the distance between the IP- and C-iGDGT distributions was 0.012 and the Significance Level was 15.6%, suggesting that the distributions from the two fractions are similar to each other.

The Sample Statistic (Global R) for the distance between the IP-, 1G- and 2G-iGDGT distributions was 0.526 and the Significance Level was 0.01%, suggesting that the dissimilarity between these three fractions is significant. The difference between every two of these three fractions was examined. The R Statistic for the IP- and 1G-iGDGTs was much less than the value for the IP- and 2G-iGDGTs or for the 1G- and 2G-iGDGTs, indicating that the IP- and 1G-iGDGT fractions have less distance than the other two pairs.

In the SIMPER test, for the IP- and 1G-iGDGT fractions, crenarchaeol and iGDGT-0 showed a great change in the average of the relative abundance (Average Abundance) and therefore had large Average Squared Distance between these two lipid fraction and great Contribution to this dissimilarity. In either of the other two pairs, the dissimilarity was contributed mainly from crenarchaeol and iGDGT-0 and minorly from iGDGTs-1 to 3 and crenarchaeol isomer. However, iGDGTs-1 to 3 and crenarchaeol isomer were observed to have great changes in the Average Abundance in each of the two pairs whereas iGDGT-0 almost exhibited no difference in the 1G- and 2G-iGDGT fractions. The low contribution of iGDGTs-1 to 3 and crenarchaeol isomer to the dissimilarity may be explained by the low proportion of these iGDGTs in the IP- and 1G-iGDGT fractions.
