# Supplementary material for: Thermoplasmatales and Methanogens: Potential Association with the Crenarchaeol Production in Chinese Soils
Source: Front Microbiol. 2017 Jun 30;8:1200. doi: 10.3389/fmicb.2017.01200 (PMC5494375; doi:10.3389/fmicb.2017.01200)
Supplement: Supplementary file 6 [file Table_3.DOCX]

Supplementary Table S3. Information on sequence number and OTU richness of taxonomy from pyrosequencing analysis in all samples. The OTU richness was based on the 99% identity level.

|  | Sequence number | | | | | | | | | | |  |  | OTU richness |
| --- | --- | --- | --- | --- | --- | --- | --- | --- | --- | --- | --- | --- | --- | --- |
|  | All phyla | | |  | Methanogens | | | |  | Thermopla-smatales | Group 1.1b A | Group 1.1b B |  | The total Archaea |
|  | Crenarc-haeota | Euryarc-haeota | Thaumar-chaeota |  | Methanob-acteriales | Methan-ocellales | Methanom-icrobiales | Methanos-arcinales |  |  |  |  |  |  |
| 20120528-27 | 0 | 575 | 493 |  | 0 | 0 | 2 | 6 |  | 34 | 0 | 492 |  | 116 |
| 20120528-36 | 0 | 756 | 78 |  | 0 | 0 | 0 | 528 |  | 1 | 0 | 34 |  | 170 |
| 20120528-37 | 0 | 676 | 1691 |  | 3 | 1 | 19 | 502 |  | 47 | 38 | 129 |  | 366 |
| 20120528-40 | 0 | 3202 | 186 |  | 47 | 25 | 425 | 2459 |  | 1 | 8 | 126 |  | 343 |
| 20120528-44 | 1 | 1968 | 1730 |  | 47 | 1 | 69 | 326 |  | 106 | 85 | 458 |  | 769 |
| 20120528-49 | 0 | 1444 | 557 |  | 1 | 0 | 0 | 6 |  | 1381 | 43 | 257 |  | 333 |
| 20120528-5 | 0 | 2551 | 4252 |  | 0 | 5 | 1 | 32 |  | 164 | 148 | 3974 |  | 906 |
| 20120528-6 | 0 | 621 | 396 |  | 0 | 2 | 0 | 1 |  | 603 | 26 | 343 |  | 210 |
| 20120814-15 | 0 | 0 | 3872 |  | 0 | 0 | 0 | 0 |  | 0 | 41 | 3814 |  | 288 |
| 20120817-33 | 0 | 71 | 3344 |  | 0 | 0 | 0 | 2 |  | 41 | 30 | 3283 |  | 327 |
| 20120817-39 | 0 | 78 | 830 |  | 0 | 0 | 0 | 0 |  | 70 | 89 | 702 |  | 378 |
| 20120817-40 | 0 | 943 | 2917 |  | 4 | 0 | 1 | 22 |  | 828 | 122 | 2746 |  | 674 |
| 20120817-49 | 0 | 39 | 4081 |  | 0 | 0 | 0 | 1 |  | 15 | 371 | 3551 |  | 458 |
| 20120817-50 | 0 | 83 | 1974 |  | 0 | 0 | 0 | 0 |  | 83 | 347 | 1537 |  | 291 |
| 20120818-52 | 0 | 531 | 438 |  | 0 | 0 | 0 | 0 |  | 529 | 3 | 426 |  | 467 |
| 20120818-57 | 0 | 1673 | 1811 |  | 0 | 0 | 0 | 1 |  | 1602 | 339 | 1258 |  | 884 |
| CM12723-11 | 0 | 394 | 782 |  | 1 | 0 | 4 | 0 |  | 382 | 40 | 708 |  | 450 |
| CM12723-28 | 0 | 24 | 2040 |  | 8 | 2 | 0 | 1 |  | 13 | 0 | 2034 |  | 185 |
| CM12723-29 | 0 | 4 | 937 |  | 0 | 0 | 0 | 0 |  | 4 | 45 | 879 |  | 145 |
| CM12723-35 | 0 | 48 | 848 |  | 0 | 0 | 0 | 0 |  | 48 | 22 | 809 |  | 178 |
| CM12723-36 | 1 | 749 | 2375 |  | 187 | 118 | 78 | 327 |  | 1 | 29 | 2152 |  | 640 |
| CM12723-37 | 0 | 4 | 858 |  | 0 | 0 | 0 | 1 |  | 1 | 79 | 733 |  | 143 |
| CM12723-4 | 0 | 62 | 1459 |  | 0 | 0 | 0 | 0 |  | 46 | 145 | 1231 |  | 407 |
| CM12723-41 | 0 | 32 | 1408 |  | 0 | 0 | 0 | 0 |  | 32 | 12 | 1377 |  | 260 |
| CM12723-46 | 0 | 197 | 605 |  | 0 | 0 | 0 | 0 |  | 190 | 33 | 563 |  | 441 |
| CM12723-51 | 0 | 7 | 969 |  | 0 | 0 | 0 | 0 |  | 5 | 8 | 930 |  | 364 |
| CM12723-60 | 0 | 377 | 781 |  | 7 | 9 | 1 | 41 |  | 315 | 38 | 690 |  | 395 |
| HN20130629-16 | 6 | 1882 | 663 |  | 46 | 41 | 527 | 1190 |  | 45 | 8 | 333 |  | 555 |
| HN20130630-29 | 0 | 1798 | 696 |  | 99 | 15 | 417 | 1042 |  | 14 | 3 | 3 |  | 551 |
| HN20130630-30 | 2 | 2151 | 79 |  | 170 | 8 | 422 | 1410 |  | 15 | 0 | 0 |  | 509 |
| HN20130630-31 | 3 | 1416 | 618 |  | 16 | 5 | 451 | 715 |  | 94 | 53 | 196 |  | 519 |
| HN20130630-32 | 2 | 1870 | 502 |  | 4 | 1 | 279 | 1474 |  | 30 | 5 | 244 |  | 446 |
| PR120109-10 | 0 | 108 | 3789 |  | 11 | 3 | 4 | 13 |  | 77 | 14 | 251 |  | 333 |
| PR120109-11 | 0 | 301 | 529 |  | 0 | 0 | 0 | 4 |  | 289 | 41 | 462 |  | 295 |
| PR120109-18 | 0 | 271 | 3457 |  | 0 | 0 | 0 | 2 |  | 247 | 64 | 3301 |  | 390 |
| PR120110-24 | 0 | 473 | 3382 |  | 2 | 4 | 0 | 9 |  | 444 | 707 | 2466 |  | 716 |
| PR120111-28 | 9 | 1494 | 555 |  | 624 | 59 | 189 | 577 |  | 15 | 10 | 150 |  | 376 |
| PR120111-31 | 0 | 2396 | 868 |  | 48 | 15 | 165 | 818 |  | 1327 | 118 | 332 |  | 769 |
| PR120112-35 | 0 | 676 | 371 |  | 7 | 4 | 2 | 7 |  | 653 | 34 | 276 |  | 426 |
| PR120112-36 | 0 | 89 | 2624 |  | 13 | 11 | 2 | 63 |  | 0 | 5 | 2182 |  | 282 |
| PR120112-41 | 0 | 1447 | 861 |  | 2 | 8 | 4 | 81 |  | 1352 | 233 | 285 |  | 475 |
| PR120601-BY-6 | 0 | 97 | 813 |  | 0 | 2 | 0 | 4 |  | 83 | 153 | 605 |  | 256 |
| PR120601-FLX-1 | 0 | 170 | 1192 |  | 30 | 2 | 2 | 11 |  | 121 | 114 | 1022 |  | 219 |
| PR120601-M1 | 2 | 473 | 2864 |  | 4 | 5 | 112 | 84 |  | 255 | 573 | 1739 |  | 724 |
| PR120601-N4 | 0 | 17 | 3980 |  | 0 | 1 | 2 | 1 |  | 12 | 370 | 3319 |  | 633 |
| PR120601-S5 | 1 | 139 | 3304 |  | 7 | 2 | 52 | 58 |  | 17 | 100 | 3060 |  | 614 |
| PR120602-BY-2 | 0 | 326 | 2415 |  | 11 | 3 | 45 | 71 |  | 179 | 287 | 2025 |  | 366 |
| QHS 12-5 | 0 | 0 | 2678 |  | 0 | 0 | 0 | 0 |  | 0 | 127 | 2448 |  | 563 |
| BHS 12-2-1 | 0 | 291 | 3551 |  | 150 | 4 | 5 | 52 |  | 1 | 34 | 3506 |  | 467 |
| BHS 12-2-3 | 0 | 1 | 2915 |  | 0 | 0 | 0 | 0 |  | 0 | 60 | 2842 |  | 276 |
