# Supplementary material for: Thermoplasmatales and Methanogens: Potential Association with the Crenarchaeol Production in Chinese Soils
Source: Front Microbiol. 2017 Jun 30;8:1200. doi: 10.3389/fmicb.2017.01200 (PMC5494375; doi:10.3389/fmicb.2017.01200)
Supplement: Supplementary file 7 [file Table_4.DOCX]

Supplementary Table S4. Sequence number of archaeal taxonomy at the family level in selected samples discussed in the main text.

|  | Halobacteri-aceae | Methanobacter-iaceae | Methanocorpusc-ulaceae | Methanomicrob-iaceae | Methanospiril-laceae | Methanosae-taceae | Methanosarci-naceae |
| --- | --- | --- | --- | --- | --- | --- | --- |
| 20120528-40 | 0 | 47 | 0 | 0 | 92 | 1199 | 1260 |
| HN20130630-32 | 0 | 4 | 0 | 15 | 3 | 199 | 27 |
| PR120111-28 | 0 | 624 | 0 | 7 | 15 | 176 | 356 |
| HN20130629-16 | 0 | 46 | 0 | 12 | 0 | 1115 | 67 |
| HN20130630-29 | 0 | 99 | 0 | 24 | 0 | 697 | 293 |
| 20120528-36 | 61 | 0 | 0 | 0 | 0 | 0 | 528 |
| PR120111-31 | 0 | 48 | 0 | 2 | 0 | 170 | 639 |
| CM12723-36 | 0 | 187 | 0 | 0 | 5 | 262 | 16 |
| 20120528-37 | 0 | 3 | 0 | 1 | 2 | 432 | 39 |
| 20120528-44 | 1 | 47 | 0 | 2 | 4 | 178 | 74 |
