# Supplementary material for: Thermoplasmatales and Methanogens: Potential Association with the Crenarchaeol Production in Chinese Soils
Source: Front Microbiol. 2017 Jun 30;8:1200. doi: 10.3389/fmicb.2017.01200 (PMC5494375; doi:10.3389/fmicb.2017.01200)
Supplement: Supplementary file 8 [file Table_5.DOCX]

Supplementary Table S5. Bivariate correlation analysis of iGDGTs-0 to 4 with archaeol in 12 samples characterized by methanogens and Thermoplasmatales in 8 samples characterized by this organism. Significant correlations are shown in bold.

|  |  | Archaeol (ng/g) | | Thermoplasmatales (%) | |
| --- | --- | --- | --- | --- | --- |
|  |  | Pearson Correlation | Sig. (2-tailed) | Pearson Correlation | Sig. (2-tailed) |
| IP-iGDGTs | iGDGT-2 (ng/g) | **0.75** | **0.005** | 0.03 | 0.942 |
| C-iGDGTs | iGDGT-0 (ng/g) | **0.60** | **0.039** | -0.50 | 0.203 |
|  | iGDGT-1 (ng/g) | **0.77** | **0.004** | -0.40 | 0.333 |
|  | iGDGT-2 (ng/g) | **0.70** | **0.011** | -0.21 | 0.625 |
| C-iGDGTs | iGDGT-2 (%) | 0 | 0.991 | **0.74** | **0.036** |
|  | iGDGT-4 (%) | -0.27 | 0.389 | **0.78** | **0.021** |
